# The Matrons' Corner

**Published:** 1887-10-01

**Authors:** 


### THE MATRONS' CORNER.

[Replies, endorsed "Matron," should be addressed to the Editor.]

S. writes: "Would a resting matron say, in reply to query in The Hospital, Sept. 17, what hospital would receive a lady as cook, as an experiment, and treat her as such, and what staff would she have as workers? The lifting of weights is the difficulty."

